# Gender, Racial, and Ethnic and Inequities in Receipt of Multiple National Institutes of Health Research Project Grants

**DOI:** 10.1001/jamanetworkopen.2023.0855

**Published:** 2023-02-28

**Authors:** Mytien Nguyen, Sarwat I. Chaudhry, Mayur M. Desai, Kafui Dzirasa, Jose E. Cavazos, Dowin Boatright

**Affiliations:** 1 Department of Immunobiology, Yale School of Medicine, New Haven, Connecticut; 2 Section of General Internal Medicine, Department of Medicine, Yale School of Medicine, New Haven, Connecticut; 3 Department of Epidemiology of Chronic Disease Epidemiology, Yale School of Public Health, New Haven, Connecticut; 4 Department of Psychiatry, Duke University School of Medicine, Durham, North Carolina; 5 South Texas Medical Science Training Program, University of Texas Health San Antonio, San Antonio; 6 Department of Emergency Medicine, New York University Grossman School of Medicine, New York

## Abstract

**Importance:**

Diversity in the biomedical research workforce is essential for addressing complex health problems. Female investigators and investigators from underrepresented ethnic and racial groups generate novel, impactful, and innovative research, yet they are significantly underrepresented among National Institutes of Health (NIH) investigators.

**Objective:**

To examine the gender, ethnic, and racial distribution of super NIH investigators who received 3 or more concurrent NIH grants.

**Design, Setting, and Participants:**

This cross-sectional study included a national cohort of NIH-funded principal investigators (PIs) from the NIH Information for Management, Planning, Analysis, and Coordination (IMPAC II) database from 1991 to 2020.

**Exposures:**

Self-identified gender, race and ethnicity, annual number of NIH grant receipt, career stage, and highest degree.

**Main Outcomes and Measures:**

Distribution of investigators receiving 3 or more research project grants, referred to as super principal investigators (SPIs), by gender, race, and ethnicity.

**Results:**

Among 33 896 investigators in fiscal year 2020, 7478 (22.01%) identified as Asian, 623 (1.8%) as Black, 1624 (4.8%) as Hispanic, and 22 107 (65.2%) as White; 21 936 (61.7%) identified as men; and 8695 (35.3%) were early-stage investigators. Between 1991 and 2020, the proportion of SPIs increased 3-fold from 704 (3.7%) to 3942 (11.3%). However, SPI status was unequal across gender, ethnic, and racial groups. Women and Black PIs were significantly underrepresented among SPIs, even after adjusting for career stage and degree, and were 34% and 40% less likely than their male and White colleagues, respectively, to be an SPI. Black women PIs were the least likely to be represented among SPIs and were 71% less likely to attain SPI status than White men PIs (adjusted odds ratio, 0.29; 95% CI, 0.21-0.41).

**Conclusions and Relevance:**

In this cross-sectional study of a national cohort of NIH-funded investigators, the gender, ethnic, and racial gaps in receipt of multiple research project grants among NIH investigators was clearly apparent and warrants further investigation and interventions.

## Key Points

**Question:**

What is the gender, racial, and ethnic diversity of elite National Institutes of Health investigators from 1991 to 2020?

**Findings:**

In this cross-sectional study, while the number of principal investigators holding 3 or more research project grants increased 3-fold between 1991 and 2020, female and Black principal investigators were significantly underrepresented in this group, even after adjusting for career stage and degree.

**Meaning:**

These results suggest that there is a growing funding gap among National Institutes of Health investigators, along with a persistent gender, race, and ethnic inequity among an elite class of SPIs. Consideration of the persistent gender, racial, and ethnic gaps in this elite class of investigators.

## Teaser

This cross-sectional study of National Institutes of Health (NIH) grants examines trends in gender, racial, and ethnic diversity among principal investigators, with a particular focus on investigators holding 3 or more research project grants.

## Introduction

Despite the benefits of diversity in scientific innovation, the distribution of National Institute of Health (NIH) funding has been historically disparate,^1^ with significant gender, racial, and ethnic inequalities in both NIH funding and success rate.^2,3,4,5,6^ In response, the NIH Working Group to the Advisory Committee and Director has made efforts in recent years to improve equity in NIH funding, leading to modest improvement in gender, racial, and ethnic representation among NIH investigators.^1^ However, little is known about gender, racial, and ethnic composition of principal investigators (PIs) who receive multiple NIH grants.

Although holding 1 research project grant is indicative of career success,^7^ academic institutions are increasingly prioritizing the recruitment and retention of principal investigators who hold multiple research project grants.^8^ A faculty member’s overall portfolio of research project grants may influence key institutional decisions regarding recruitment, salary, tenure, promotion, and resource allocations, as well as national policy decisions on research funding.^8,9^ Despite the significant power and resources held by investigators with multiple, simultaneous research grants (hereafter referred to as super principal investigators [SPI]), gender, racial, and ethnic composition of SPIs is currently unknown.

To evaluate gender, racial, and ethnic diversity of SPIs, we examined the distribution of SPIs over time using a national database of NIH investigators from 1991 to 2020. We also examined the likelihood of being an SPI by intersectional gender and racial or ethnic minority identity.

## Methods

### Data Source

Data were obtained for principal investigator–specific research project grants from the NIH Information for Management, Planning, Analysis, and Coordination (IMPAC II) database for fiscal years 1985 to 2020.^1^ The IMPAC II is a database maintained by the NIH and is provided with limited use. Research project grants included grants with the following activity codes: DP1, DP2, DP3, DP4, DP5, P01, PN1, PM1, R00, R01, R03, R15, R21, R22, R23, R29, R33, R34, R35, R36, R37, R61, R50, R55, R56, RC1, RC2, RC3, RC4, RF1, RL1, RL2, RL9, RM1, UA5, UC1, UC2, UC3, UC4, UC7, UF1, UG3, UH2, UH3, UH5, UM1, UM2, U01, U19, and U34. Grants awarded under the American Recovery and Reinvestment Act of 2009 (ARRA) and supplemental COVID-19 appropriations were excluded. Funding dollars were adjusted for inflation to 2019 US dollars using the Biomedical Research and Development Price Index. Patients or the public were not involved in the design, conduct, reporting, or dissemination plans of our research. Our analyses were conducted according to the Strengthening the Reporting of Observational Studies in Epidemiology (STROBE) reporting guidelines and were deemed exempt by the Yale University institutional review board.

### Demographic Variables

PIs’ gender, race, and ethnicity were self-reported by the faculty applying for NIH grant funding. PIs with unknown or withheld gender identity (14 291 [1.8%]) were excluded from regression analyses. We examined trends in research project grants from 1991 to 2020 due to a significant portion of missing racial and ethnic data on investigators prior to 1991. Racial categories included American Indian or Alaska Native, Asian, Black or African American, Native Hawaiian or other Pacific Islander, White, more than 1 race, unknown, or withheld. Ethnicity categories included Hispanic, Not Hispanic, unknown, or withheld. Racial and ethnic identities were combined into the following categories: Asian, Black, Hispanic, White, and other (which included American Indian or Alaska Native, Native Hawaiian or other Pacific Islander, more than 1 race, unknown, or withheld). PIs who reported Hispanic ethnicity were categorized as Hispanic regardless of racial identity.

### Defining SPIs

We defined SPIs as principal investigators holding 3 or more concurrent active research project grants in a given fiscal year (approximately the top 10% of all NIH principal investigators in 2020). Unadjusted rates of being an SPI were determined by calculating the proportion of each gender, ethnic and racial, or intersectional group that were SPIs vs non-SPIs.

The IMPAC II data set comprise both small and large dollars research project grants. To determine the robustness of utilizing number of grants to define SPI, we compared median and interquartile range (IQR) of total funding dollars received among SPIs and non-SPIs across demographic groups for fiscal year 2020.

### Statistical Analysis

Nonparametric *t* tests and Kruskal-Wallis tests with posthoc Dunn correction for multiple comparisons were used to determine significance between median funding dollar amounts across groups. Multivariable logistic regression was used to determine the relative odds of women and investigators from underrepresented racial and ethnic groups of being an SPI compared with men and White investigators, respectively. Covariates include PI’s highest degree and career stage. Degree was defined as MD, MD/PhD, PhD, or other degrees. PI’s career stage was approximated using investigator’s age, categorized as early (age under 46 years), middle (age 46 to 58 years), and late (age above 58 years), as described previously.^1^ Finally, we included 3 time periods that delineate significant changes in the NIH budget: 1991-1998 (phase 1) before the first budget increase, 1999-2014 (phase 2) between the first and second budget increase, and 2015-2020 (phase 3) after the second budget increase, and tested the interaction between phase and gender, ethnic, and racial identities to determine whether the relative odds of being an SPI for disadvantaged groups (eg, women, Black, and Hispanic) have changed over time. Adjusted percentage of SPI investigators within each combined subgroup of gender, ethnic, and racial identity was determined from the fully adjusted logistic models. Statistical tests were 2-sided with type 1 error rate of 0.05. All analyses were performed using Stata version 16.1 (Stata Inc).

## Results

### Trends in NIH SPIs

In fiscal year 1991, among 18 820 investigators, 1187 (6.4%) identified as Asian, 100 (0.5%) as Black, 320 (1.7%) as Hispanic, and 14 630 (77.7%) as White; 13 821 (80.0%) identified as men; and 9124 (52.8%) were early-stage investigators. In comparison, in fiscal year 2020, among 33 896 investigators, 7478 (22.1%) identified as Asian, 623 (1.8%) as Black, 1624 (4.8%) as Hispanic, and 22 107 (65.2%) as White; 21 936 (61.7%) identified as men; and 8695 (35.3%) were early-stage investigators (Table).

**Table.** Characteristics of National Institutes of Health–Funded Investigators by Super Principal Investigator (SPI) Status, 2020

Characteristics	Investigators, No. (%)			*P* value
	All (N = 33 896)	Non-SPI (n = 29 989)	SPI (n = 3907)	
Ethnicity and race				
Asian	7478 (22.1)	6523 (21.8)	955 (24.4)	<.001
Black	623 (1.8)	588 (2.0)	35 (0.9)	
Hispanic	1624 (4.8)	1465 (4.9)	159 (4.1)	
Other^a^	2064 (6.1)	1861 (6.2)	203 (5.2)	
White	22 107 (65.2)	19 552 (65.2)	2555 (65.4)	
Gender				
Men	21 936 (64.7)	19 068 (63.6)	2868 (73.4)	<.001
Women	11 960 (35.3)	10 921 (36.4)	1039 (26.6)	
Degree				
PhD	24 371 (71.9)	21 728 (72.5)	2643 (67.6)	<.001
MD/PhD	3599 (10.6)	2999 (10.0)	600 (15.4)	
MD	5274 (15.6)	4623 (15.4)	651 (16.7)	
Other	652 (1.9)	639 (2.1)	13 (0.3)	
Career stage				
Early (<46 y)	10 386 (30.6)	9546 (31.8)	840 (21.5)	<.001
Middle (46-58 y)	8395 (24.8)	7294 (24.3)	1101 (28.2)	
Late (>58 y)	12 969 (38.3)	11 228 (37.4)	1741 (44.6)	
Unknown	2146 (6.3)	1921 (6.4)	225 (5.8)	
**Combined identity subgroups**				
Asian				<.001
Men	5088 (15.0)	4356 (14.5)	732 (18.7)	
Women	2390 (7.1)	2167 (7.2)	223 (5.7)	
Black				
Men	334 (1.0)	311 (1.0)	23 (0.6)	
Women	289 (0.9)	277 (0.9)	12 (0.3)	
Hispanic				
Men	1021 (3.0)	902 (3.0)	119 (3.0)	
Women	603 (1.8)	563 (1.9)	40 (1.0)	
Other^a^				
Men	1423 (4.2)	1268 (4.2)	155 (4.0)	
Women	641 (1.9)	593 (2.0)	48 (1.2)	
White				
Men	14 070 (41.5)	12 231 (40.8)	1839 (47.1)	
Women	8037 (23.7)	7321 (24.4)	716 (18.3)	

^a^ Other included American Indian or Alaska Native, Native Hawaiian or other Pacific Islander, more than 1 race, unknown, or withheld.

From 1991 to 2020, the total number of NIH PIs increased 1.8-fold from 18 820 to 34 936 (eFigure 1 in Supplement 1), which corresponds to a spending increase from $11.9 billion to $22.4 billion. Notably, there were 2 years in which the NIH budget increased substantially, in 1998 and 2015.

**Figure 1.**
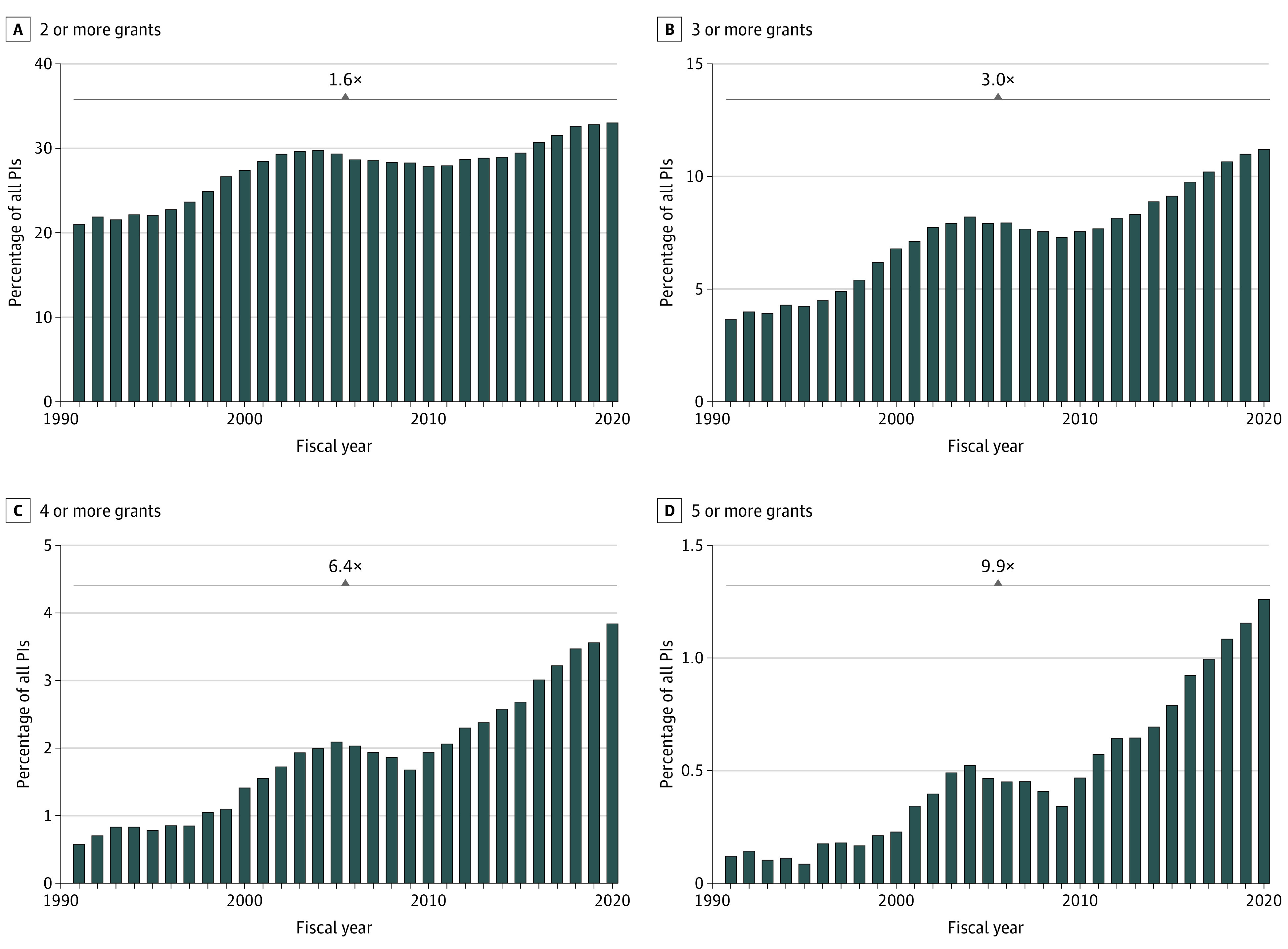
Proportion of Principal Investigators (PIs) With Active Concurrent Research Project Grants From 1991-2020 Super principal investigators (SPIs) included all PIs with 3 or more research project grants (approximately the top 10% of PIs in 2020).

We next examined trends in PIs holding multiple grants by summarizing the proportion of all PIs who held multiple active grants in a given fiscal year at 4 levels: PIs who held 2 or more active grants, 3 or more grants, 4 or more grants, or 5 or more grants (Figure 1). Since 1991, the proportion of PIs who held multiple grants has increased overall, with major inflection points occurring when there was an increase in the NIH budget in 1998 and 2015). Between 1991 and 2020, the percentage of PIs with 2 or more research project grants increased by 60% (from 21.2% to 33.2%), while the percentage of PIs with 3 or more research project grants increased 3-fold (from 3.7% to 11.3%). The percentage of PIs with 4 or more research project grants increased more than 6-fold (from 0.6% to 3.8%), and the percentage of PIs with 5 or more research project grants increased 10-fold (from 0.1% to 1.2%).

The number of PIs with 3 or more research project grants grew at a rate that outpaced the baseline increase in NIH investigators (3.0-fold vs 1.8-fold; *P* < .001). This SPI cohort of PIs with 3 or more concurrent active research project grants represented 10% of all NIH-funded investigators in the past 5 years. Funding allocation to SPIs increased more than 2-fold from 12.7% in 1991 to 28.0% in 2020 (eFigure 2A in Supplement 1). In 2020, SPIs, who comprise 11.3% of all PIs, received 28.0% of federal NIH research funding, with a median (IQR) annual total research funding of $1.42 million ($1.08-$2.05 million) per PI compared with $0.38 million ($0.25-$0.62 million) per PI among non-SPI (*P* < .001) (eFigure 2B in Supplement 1).

To determine the robustness of our findings, we performed a sensitivity analysis using total grant dollars across gender and ethnic and racial identities. Across all gender, ethnic, and racial groups, SPIs received a significant higher median (IQR) annual research project grant funding compared with non-SPIs (men: non-SPIs, $0.39 million [$0.26-$0.62 million] vs SPIs, $1.30 million [$0.95-$1.73 million]; women: non-SPIs, $0.38 million [$0.24-$0.61 million] vs SPIs, $1.30 million [$0.94-$1.74 million]; *P* < .001) (eFigure 3A and 3B in Supplement 1). There was no difference in annual median research funding dollars for men and women SPIs ($1.30 million for both men and women SPIs; *P* > .99), or across ethnic and racial groups ($1.30 million [$0.95-$1.77 million] for White SPIs, $1.20 million [$0.92-$1.61 million] for Asian SPIs, $1.20 million [$0.89-$1.70 million] for Hispanic SPIs, and $1.50 million [$1.00-$1.84 million] for Black SPIs; Dunn-corrected *P* > .05 for all comparisons) (eFigure 1B, eFigure 3 in Supplement 1).

### Representation of Women and Black NIH Investigators Among SPIs

**Figure 2.**
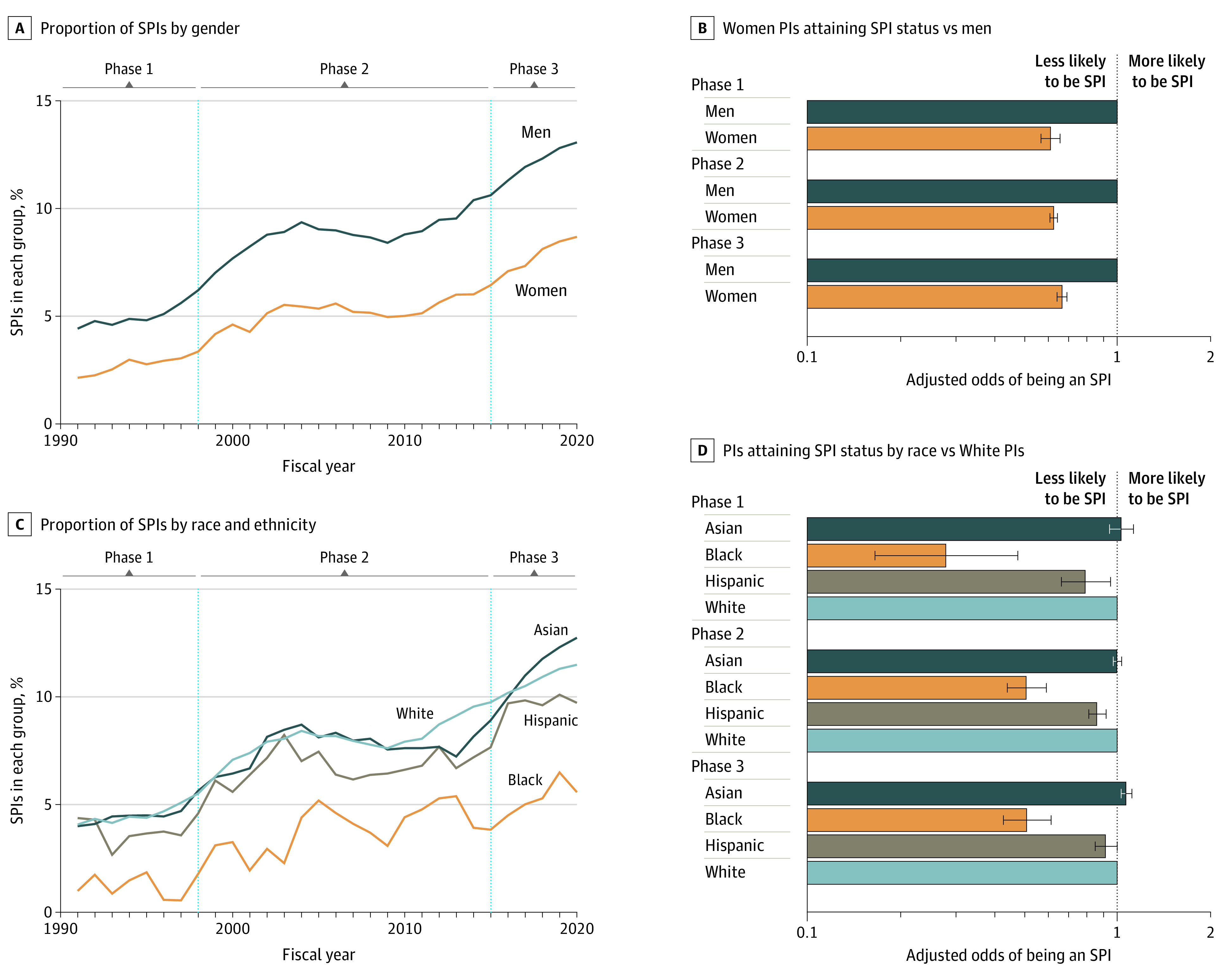
Gender, Ethnic, and Racial Diversity Among SPIs Error bars indicate 95% CIs. In panel B, odds ratios adjusted for career stage (early, middle, and late) and degree; in panel D, odds adjusted for career stage and degree.

Next, we examined gender composition of SPIs (Figure 2A and Figure 2B). In 1991, 2.1% of women and 4.4% of men were SPIs. By 2020, these numbers increased to 8.7% and 13.1% for women and men, respectively (Figure 2A; eTable 1 in Supplement 1). After adjusting for degree and career stage, women PIs had 40%, 38%, and 34% lower odds than men to attain SPI status in phases 1, 2, and 3, respectively (Figure 2B; eTable 1 in Supplement 1). While the interaction analysis between time phase and gender revealed that the relative disadvantage of women attaining SPI status has diminished over time (*P* = .003), women continue to have significantly lower odds of being an SPI than men.

There was similar inequity in ethnic and racial representation among SPIs. In 1992, 4.1%, 4.0%, 4.4%, and 1.0% of White, Asian, Hispanic, and Black PIs, respectively, were SPIs (Figure 2C). Although these proportions increased for all ethnic and racial groups over time, the proportion of Black PIs having SPI status remained significantly lower than White PIs in phase 3 (5.6% Black vs 11.5% White; *P* < .001). Interaction analysis between time phase and ethnic and racial identity indicated that the relative odds of being an SPI for PIs of color (eg, Asian, Hispanic, and Black) significantly changed over time (*P* < .001). In phase 1, after adjusting for degree and career stage, compared with White PIs, Asian PIs were as likely to be an SPI (adjusted odds ratio [aOR], 1.03; 95% CI, 0.94-1.13), and Black and Hispanic PIs were less likely to be an SPI (Black: aOR, 0.28; 95% CI, 0.16-0.47; Hispanic: aOR, 0.79; 95% CI, 0.66-0.95) (Figure 2D; eTable 1 in Supplement 1). By phase 3, Asian PIs were significantly more likely than White PIs to be an SPI (aOR, 1.07; 95% CI, 1.03-1.11), while Hispanic PIs were as likely as White PIs to be an SPI (aOR, 0.92; 95% CI, 0.84-1.00). Although the likelihood of Black PIs having SPI status increased over time, Black PIs were still half as likely as White PIs to be SPIs in phase 3 (aOR, 0.51; 95% CI, 0.42-0.61) (Figure 2D; eTable 1 in Supplement 1).

### Black Women PIs Least Likely to Be SPIs

We examined the intersections between gender and ethnic and racial identity among SPIs over time. Between 1991 and 2020, the proportion of SPIs among White, Asian, and Hispanic men increased at a higher rate compared with Black men and all women (eFigure 4 in Supplement 1). In 2020, while 13.1% of White men PIs were SPIs, only 6.8% and 4.1% of Black men and women PIs were SPIs, respectively (*P* < .001).

In phase 1, after adjusting for degree and career stage, compared with White men, Asian and Hispanic men were as likely to be an SPI (Asian men PIs: aOR, 1.09; 95% CI, 0.99-1.20; Hispanic men PIs: aOR, 0.84; 95% CI, 0.70-1.02) while Black men were significantly less likely to be an SPI (aOR, 0.33; 95% CI, 0.19-0.58) (eFigure 4, eTable 2 in Supplement 1). Compared with White men PIs, all women PIs across ethnic and racial groups were less likely to be an SPI in phase 1, with Black women PIs having the largest disadvantage (White women PIs: aOR, 0.63; 95% CI, 0.58-0.68; Asian women PIs: aOR, 0.42; 95% CI, 0.32-0.55; Hispanic women PIs: 0.26; 95% CI, 0.13-0.52; Black women PIs: 0.05; 95% CI, 0-0.39) (eFigure 4, eTable 2 in Supplement 1).

**Figure 3.**
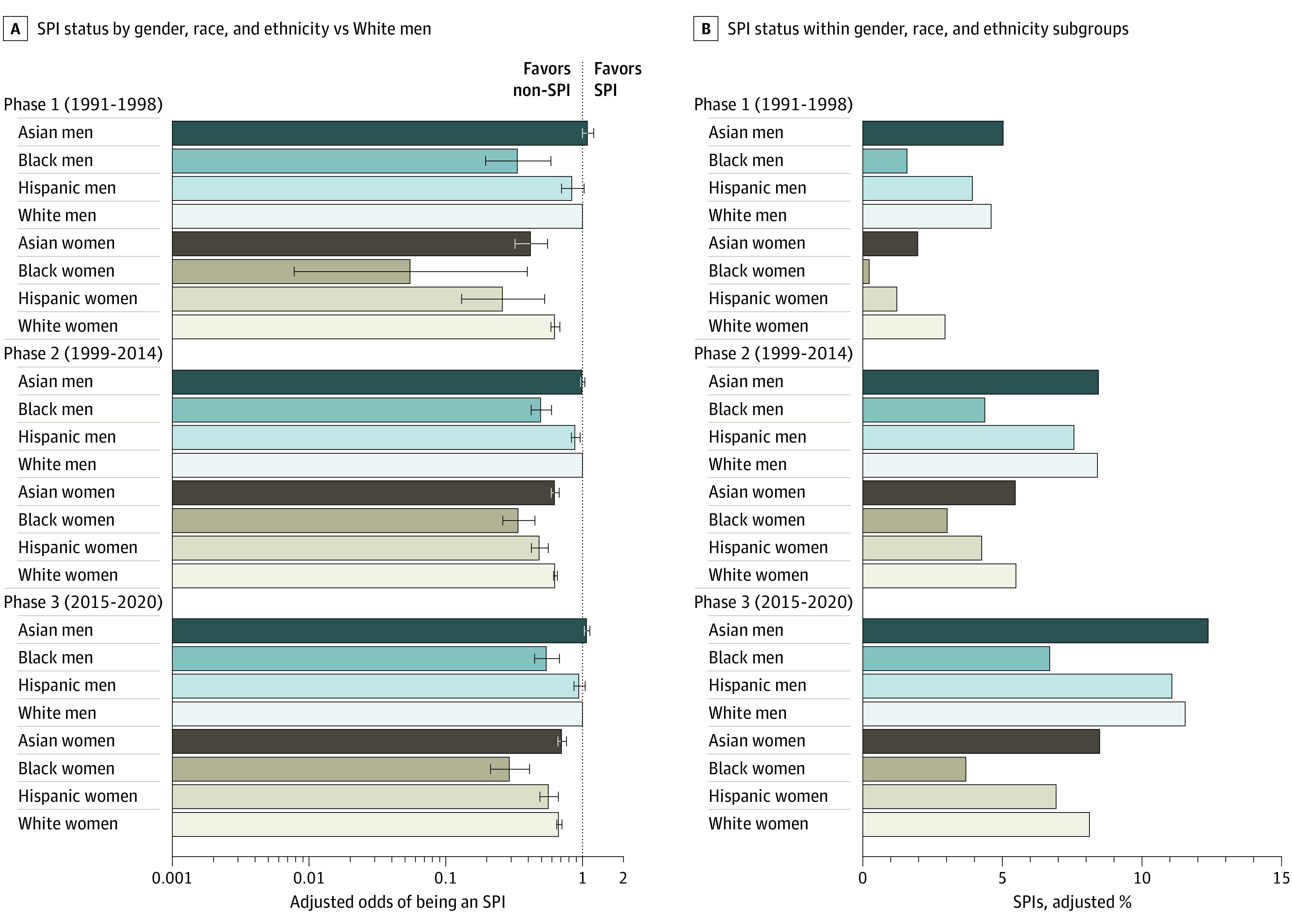
Proportion of SPIs by Gender, Ethnic, and Racial Intersectional Subgroups Error bars indicates 95% CIs. Odds ratios were adjusted for career stage and degree.

By phase 3, compared with White men, Asian men were more likely (aOR, 1.08; 95% CI, 1.03-1.13), and Hispanic men were as likely (aOR, 0.95; 95% CI, 0.86-1.04) to be an SPI, while Black men remained less likely to be an SPI (aOR, 0.55; 95% CI, 0.44-0.67) (Figure 3). Compared with White men, White, Asian, Hispanic and Black women were 33%, 29%, 43%, and 71% less likely to be an SPI in phase 3. Although interaction analysis revealed that the relative odds of being an SPI improved over time for Black men and for women across all ethnic and racial groups (*P* < .001), these groups remained significantly less likely than White men to be an SPI in phase 3 (Figure 3). Remarkably, even though the relative odds of Black women being an SPI improved after the first NIH budget increase (phase 1: aOR, 0.05; 95% CI, 0.00-0.39; phase 2: aOR, 0.34; 95% CI, 0.26-0.44), these odds have not changed after the most recent NIH budget increase (aOR, 0.29; 95% CI, 0.21-0.41) (Figure 3).

## Discussion

In this study, we found that an elite class of principal investigators who held 3 or more grants grew in number over the past 30 years. The proportion of SPIs among all PIs increased 3-fold from 3.7% in 1991 to 11.3% in 2020. Moreover, the compositional diversity of SPIs was not equitable across gender and ethnic and racial groups. Even after adjusting for career stage and degree, women and Black PIs were significantly less likely to have SPI status compared with White PIs. Black women were most disparately underrepresented among SPIs, with White men PIs being more than 3-fold more likely to be an SPI compared with Black women.

The rise in the percentage of SPIs among all investigators and the concurrent ethnic and racial disparity among SPIs is concerning. Despite evidence of diminishing returns on investment for PIs receiving greater than $600 000 per year in funding,^10,11^ data suggest that NIH dollars are increasingly concentrated among a small proportion of investigators. This led NIH leadership to consider capping NIH funding to 3 R01-equivalent grants per investigator,^12^ although this policy was never implemented across NIH institutes and centers. Given the well-documented benefits of diversity among investigative teams, ethnic and racial disparities among PIs and SPIs could limit scientific impact and innovation,^13,14,15,16^ posing a substantial threat to the success of the US biomedical research enterprise.

While the cause of the gender, ethnic, and racial gap in SPI status reported in this study is likely multifactorial, disparities in mentorship available to Black and women faculty may contribute to this gap.^17^ Mentorship not only guides early career faculty on a path to success but also exposes faculty to a network of peers that will facilitate collaborations and support.^18,19,20^ Black and women scientists are less likely than White and men scientists to be mentored by high impact senior mentors,^21^ and therefore less likely to acquire the scientific network, tacit knowledge, and sponsorship that are inherently required for securing grants. Furthermore, even when mentored by senior faculty, bias and racism may affect the relationship that Black and women faculty have with their mentors, resulting in negative mentoring that harms women and faculty of color.^22,23,24^

Patterns of grant submission may also influence the disparities in SPI status by gender, race, and ethnicity described in this study. Higher frequency grant submission and resubmission have been linked to funding success,^25^ and prior studies have reported that women, in aggregate, and Black faculty submit fewer grants than their counterparts.^26,27^ Investment in both early and mid-career meaningful mentorship initiatives for Black and women faculty will be essential to improve funding longevity and reduce the inequitable ethnic and racial distribution of NIH funding allocated to first-time PIs and among more established SPIs.^28^ Such programs may include expansion of diversity supplements for early-career faculty, developing mentoring networks for female and Black faculty,^29,30,31^ and incentivizing diverse team-based science through additional emphasis in program grants and multiple principal investigator awards.

While programs to support grant submission and resubmission are critical, this intervention will likely remain insufficient to address the disparities reported in this study as prior research has shown that Black faculty receive lower scores and are less likely to be funded after grant resubmission than their White counterparts, even after controlling for training record, prior award, and publication history.^26,27^ Therefore, women and faculty from underrepresented ethnic and racial groups face disparity at 2 levels—initial application^32^ and reapplication^26,27^—suggesting a worrying trend of inequitable resource allocation at all career stages. These data suggest that structural interventions may be necessary to address bias in grant assessment, such as diversifying members of NIH study sections and program staff,^33^ which in 2021 was 33.8% female, 2.3% Black, and 4.5% Hispanic.^34^ In addition to increasing diversity in study sections, the NIH could consider promoting bias training and education among reviewers.

Despite historical struggles with diversity among PIs, the NIH has introduced several interventions to address funding disparities in recent years. In 2021, the NIH established the UNITE initiative to address systemic racism in the NIH and biomedical researcher workforce.^35^ The committees operating under the UNITE initiative have made considerable efforts to increase workforce diversity and reduce disparities in research funding, such as increasing funding to NIH institutes that receive a higher percentage of applications from female and faculty from underrepresented ethnic and racial groups (eg, National Institute on Minority Health and Health Disparities), increasing support to minority-serving institutions, and implementation of the Faculty Institutional Recruitment for Sustainable Transformation (FIRST) funding opportunity to support the recruitment of diverse faculty cohorts.^36^

Moving forward, the UNITE initiative and the NIH could consider structural changes in the grant review process to assess the ethnic and racial diversity of investigators listed in grant applications. Diverse teams are more innovative and produce higher quality research than homogenous teams,^13,15,37^ and including an investigator team diversity score could represent an evidence-based metric to promote high-impact science. Additionally, the NIH could incorporate an assessment of the institutional climate of equity and inclusion as a component of a grant application’s scoring criteria. Measures of equity and inclusion could include the institution’s compositional faculty diversity and equity in promotion and salary.

Other structural reforms may include changes to timing of funding deadlines. Request for Application (RFA) and Funding Opportunity Announcement (FOA) for NIH grants are often released shortly before the submission deadline. Women, Hispanic, and Black scientists are less likely to have access to strong research networks and mentorship^22,23^ and are frequently overtasked with unpaid and unrewarded administrative duties that can be detrimental to their research and career success,^38,39^ including meeting grant submission deadlines. Providing more time between FOA and RFA release and submission deadline would allow women and faculty from underrepresented ethnic and racial groups time and resources to build and submit their grant applications.

### Limitations

Our study had several limitations. This study examined the likelihood of being an SPI given that an investigator had already received NIH funding and does not account for submission behavior differences across demographic groups nor the environmental support (such as research funding available at the institution). These factors may affect the gender, racial, and ethnic disparities described and are important to examine to inform future policies and interventions. Furthermore, our study included funding to contact principal investigators and does not include delineation of whether a grant has multiple PIs. The multiple PI approach is critical for team-based science and can play an important role to improve diversity, as well as mentoring for young women and underrepresented faculty. In addition, a small percentage of PIs withheld their ethnic or racial identity and some ethnic and racial groups, such as Alaska Native, Native American, Hawaiian Native, and Pacific Islanders, were too small for analysis. The small number of Indigenous investigators reflected systemic marginalization across biomedicine and society. More attention should be paid to promote and enhance biomedical research funding to Indigenous investigators, as well as researchers with other marginalized identities such as socioeconomic disadvantage and faculty with disability.^40^ Lastly, this study focused on NIH-funded investigators and have limited generalizability to other federal and nonfederal funding agencies, such as the National Science Foundation,^41^ for which further study is needed.

## Conclusions

In this cross-sectional study of NIH investigators from 1991 to 2020, we found a growing gap among NIH investigators that created a cohort of highly funded NIH investigators. Importantly, there were persistent gender, ethnic, and racial inequities among this elite class of SPIs. As the NIH develops critical initiatives and reforms to promote equity among its investigators, consideration of the persistent gender and ethnic and racial gaps in this elite class and the influence they have is critical for meaningful reform.

## Notes

Supplement 1. **eFigure 1.** Total Number of NIH-funded Principal Investigators (PIs) and NIH Budget Spending From 1991-2020 **eFigure 2.** Distribution of Funding Between SPI and Non-SPI **eFigure 3.** Median Research Project Grant Funding for Fiscal Year 2020 Among SPIs and Non-SPIs **eFigure 4.** Distribution of SPI Status by Gender-Racial/Ethnic Identity **eTable 1.** Unadjusted and Adjusted Multiple Logistic Regression of Being an SPI Over Time **eTable 2.** Unadjusted and Adjusted Multiple Logistic Regression of Being an SPI Over Time by Gender-Racial/Ethnic Intersectional Identity

Supplement 2.
Data Sharing Statement
