# Supplementary material for: Gender, Racial, and Ethnic and Inequities in Receipt of Multiple National Institutes of Health Research Project Grants
Source: JAMA Netw Open. 2023 Feb 28;6(2):e230855. doi: 10.1001/jamanetworkopen.2023.0855 (PMC9975935; doi:10.1001/jamanetworkopen.2023.0855)
Supplement: Supplement 1. — eFigure 1. Total Number of NIH-funded Principal Investigators (PIs) and NIH Budget Spending From 1991-2020 eFigure 2. Distribution of Funding Between SPI and Non-SPI eFigure 3. Median Research Project Grant Funding for Fiscal Year 2020 Among SPIs and Non-SPIs eFigure 4. Distribution of SPI Status by Gender-Racial/Ethnic Identity eTable 1. Unadjusted and Adjusted Multiple Logistic Regression of Being an SPI Over Time eTable 2. Unadjusted and Adjusted Multiple Logistic Regression of Being an SPI Over Time by Gender-Racial/Ethnic Intersectional Identity [file jamanetwopen-e230855-s001.pdf]

Supplemental Online Content

Nguyen M, Chaudhry SI, Desai MM, Dzirasa K, Cavazos JE, Boatright D. Gender, racial, and ethnic inequities in receipt of multiple National Institutes of Health research project grants. *JAMA Netw Open.* 2023;6(2):e230855. doi:10.1001/jamanetworkopen.2023.0855

eFigure 1. Total Number of NIH-funded Principal Investigators (PIs) and NIH Budget Spending From 1991-2020

eFigure 2. Distribution of Funding Between SPI and Non-SPI

eFigure 3. Median Research Project Grant Funding for Fiscal Year 2020 Among SPIs and Non-SPIs

eFigure 4. Distribution of SPI Status by Gender-Racial/Ethnic Identity

eTable 1. Unadjusted and Adjusted Multiple Logistic Regression of Being an SPI Over Time

eTable 2. Unadjusted and Adjusted Multiple Logistic Regression of Being an SPI Over Time by Gender-Racial/Ethnic Intersectional Identity

This supplemental material has been provided by the authors to give readers additional information about their work.

© 2023 Nguyen M et al. *JAMA Network Open.*

eFIGURE 1

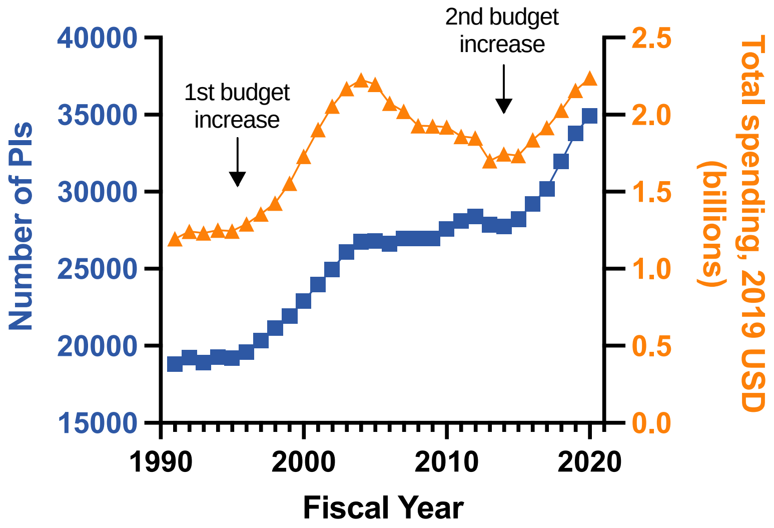

eFigure 1. Total number of NIH-funded principal investigators (PIs) and NIH budget spending from 1991-2020. Number of unique PIs (blue) with active NIH RPGs and total NIH budget spending (orange) from 1991 to 2020.

© 2023 Nguyen M et al. *JAMA Network Open.*

eFIGURE 2

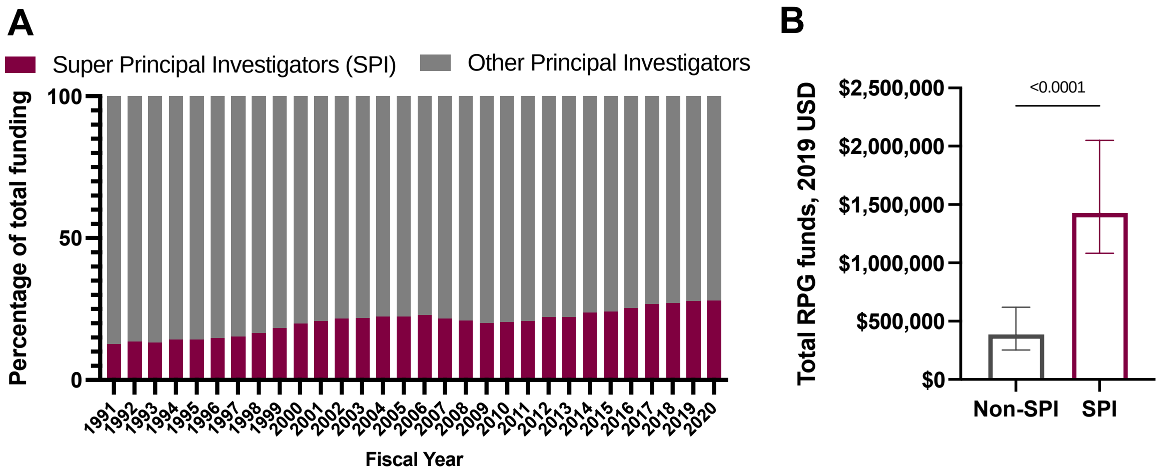

eFigure 2. Distribution of funding between SPI and non-SPI. A) Percentage of total NIH funds to SPI (three or more RPGs) and other PIs from 1991-2020. **B**) Median and interquartile range (IQR) of total NIH funds across active RPGs among SPI and other PIs in fiscal year 2020.

© 2023 Nguyen M et al. *JAMA Network Open.*

eFIGURE 3

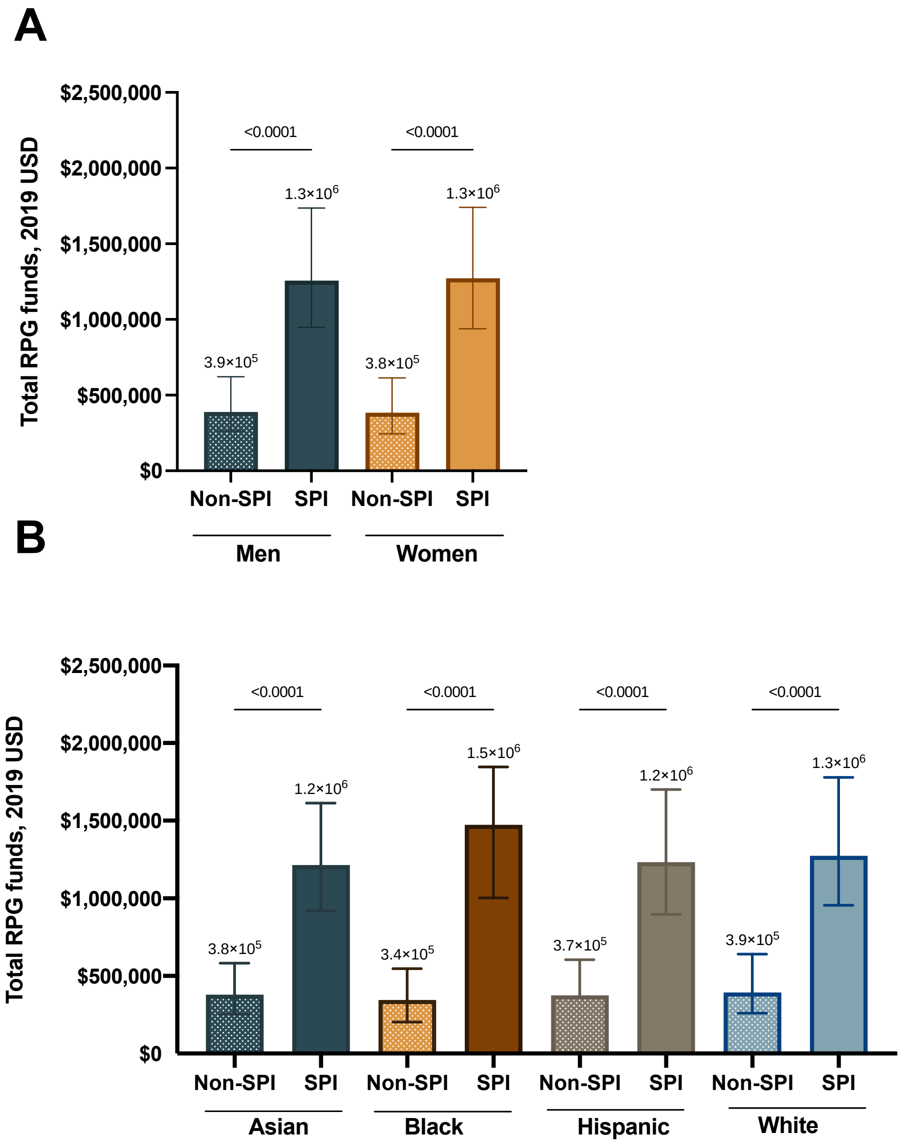

eFigure 3. Median research project grant funding for fiscal year 2020 among SPIs and non-SPIs. Median total research project grant funding in fiscal year 2020 among non-SPI and SPI by **A**) gender and **B**) racial and ethnic identity. Error bars indicate interquartile range. Significant comparisons between groups are shown using Kruskal-Wallis non-parametric test with Dunn's correction for multiple comparisons.

© 2023 Nguyen M et al. *JAMA Network Open.*

eFIGURE 4

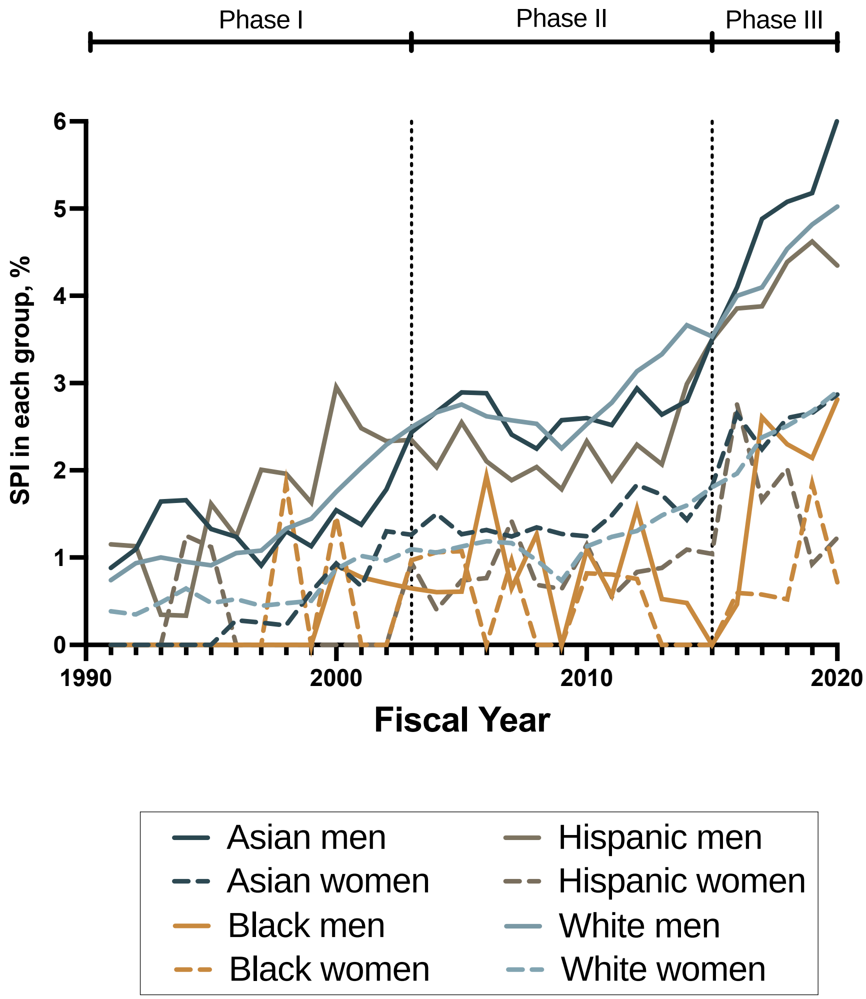

eFigure 4. Distribution of SPI status by gender, racial, and ethnic identity. Proportion of women and men PIs by gender, racial, and ethnic identity with three or more RPGs.

© 2023 Nguyen M et al. *JAMA Network Open.*

eTable 1. Unadjusted and adjusted multiple logistic regression of being a SPI over time.

	Unadjusted Odds Ratio			Adjusted Odds Ratio		
	**Phase I**	**Phase II**	**Phase III**	**Phase I**	**Phase II**	**Phase III**
**Gender**						
Men	*Ref*	*Ref*	*Ref*	*Ref*	*Ref*	*Ref*
Women	0.53 (0.50-0.57)	0.57 (0.55-0.59)	0.62 (0.59-0.64)	0.60 (0.56-0.65)	0.62 (0.60-0.64)	0.66 (0.64-0.68)
**Racial and ethnic identity**						
Asian	1.00 (0.92-1.09)	0.96 (0.93-0.99)	1.07 (1.03-1.11)	1.03 (0.94-1.13)	1.00 (0.97-1.03)	1.07 (1.03-1.11)
Black	0.25 (0.15-0.43)	0.47 (0.41-0.54)	0.47 (0.39-0.56)	0.28 (0.16-0.47)	0.51 (0.44-0.59)	0.51 (0.42-0.61)
Hispanic	0.82 (0.68-0.98)	0.83 (0.78-0.89)	0.88 (0.82-0.96)	0.79 (0.66-0.95)	0.86 (0.81-0.92)	0.92 (0.85-1.00)
Other	0.88 (0.79-0.99)	0.79 (0.75-0.84)	0.83 (0.77-0.89)	1.03 (0.94-1.13)	0.82 (0.77-0.86)	0.88 (0.82-0.95)
White	Ref	Ref	Ref	Ref	Ref	Ref
**Degree**						
PhD	*Ref*	*Ref*	*Ref*	*Ref*	*Ref*	*Ref*
MD/PhD	1.91 (1.78-2.05)	1.80 (1.75-1.86)	1.68 (1.6-1.76)	1.84 (1.71-1.98)	1.66 (1.61-1.72)	1.49 (1.42-1.56)
MD	1.40 (1.32-1.48)	1.36 (1.32-1.4)	1.18 (1.13-1.23)	1.29 (1.22-1.37)	1.22 (1.19-1.26)	1.08 (1.03-1.13)
Other	0.53 (0.36-0.78)	0.35 (0.29-0.43)	0.19 (0.14-0.25)	0.58 (0.39-0.84)	0.36 (0.30-0.44)	0.20 (0.15-0.26)
**Age**						
Early (<46 years-old)	*Ref*	*Ref*	*Ref*	*Ref*	*Ref*	*Ref*
Mid (46-58 years-old)	2.25 (2.13-2.38)	2.08 (2.02-2.14)	1.82 (1.74-1.90)	2.20 (2.08-2.33)	2.00 (1.94-2.05)	1.70 (1.63-1.77)
Late (>58 years-old)	2.08 (1.91-2.26)	2.04 (1.97-2.11)	1.87 (1.78-1.96)	1.81 (1.66-1.97)	1.92 (1.86-1.99)	1.72 (1.64-1.80)
Unknown	1.74 (1.56-1.95)	1.84 (1.75-1.93)	1.42 (1.31-1.53)	1.89 (1.69-2.11)	1.81 (1.73-1.90)	1.42 (1.32-1.53)

© 2023 Nguyen M et al. *JAMA Network Open.*

eTable 2. Unadjusted and adjusted multiple logistic regression of being a SPI over time by gender, racial, and ethnic intersectional identity.

	Unadjusted Odds Ratio			Adjusted Odds Ratio		
	**Phase I**	**Phase II**	**Phase III**	**Phase I**	**Phase II**	**Phase III**
**Gender, racial, and ethnic identity**						
Asian men	1.05 (0.95-1.15)	0.94 (0.91-0.97)	1.05 (1.01-1.10)	1.09 (0.99-1.20)	1.00 (0.96-1.03)	1.08 (1.03-1.13)
Asian women	0.38 (0.28-0.49)	0.56 (0.53-0.60)	0.66 (0.62-0.71)	0.42 (0.32-0.55)	0.63 (0.58-0.67)	0.71 (0.66-0.76)
Black men	0.32 (0.18-0.56)	0.49 (0.41-0.58)	0.53 (0.43-0.65)	0.33 (0.19-0.58)	0.50 (0.42-0.59)	0.55 (0.44-0.67)
Black women	0.04 (0.00-0.34)	0.29 (0.22-0.39)	0.25 (0.18-0.35)	0.05 (0.00-0.39)	0.34 (0.26-0.44)	0.29 (0.21-0.41)
Hispanic men	0.88 (0.73-1.06)	0.88 (0.82-0.94)	0.92 (0.84-1.01)	0.84 (0.70-1.02)	0.89 (0.82-0.95)	0.95 (0.86-1.04)
Hispanic women	0.21 (0.10-0.43)	0.42 (0.37-0.49)	0.51 (0.44-0.60)	0.26 (0.13-0.52)	0.48 (0.42-0.56)	0.57 (0.48-0.66)
Other men	0.84 (0.75-0.95)	0.81 (0.76-0.85)	0.83 (0.77-0.91)	0.81 (0.71-0.91)	0.85 (0.80-0.89)	0.91 (0.84-0.99)
Other women	0.59 (0.44-0.78)	0.36 (0.32-0.42)	0.45 (0.39-0.53)	0.64 (0.48-0.85)	0.42 (0.37-0.48)	0.53 (0.46-0.63)
White men	*Ref*	*Ref*	*Ref*	*Ref*	*Ref*	*Ref*
White women	0.55 (0.51-0.60)	0.58 (0.56-0.59)	0.63 (0.6-0.65)	0.63 (0.58-0.68)	0.63 (0.61-0.65)	0.67 (0.64-0.70)
**Degree**						
PhD	*Ref*	*Ref*	*Ref*	*Ref*	*Ref*	*Ref*
MD/PhD	1.91 (1.78-2.05)	1.80 (1.75-1.86)	1.68 (1.6-1.76)	1.84 (1.71-1.98)	1.66 (1.61-1.72)	1.49 (1.42-1.56)
MD	1.40 (1.32-1.48)	1.36 (1.32-1.4)	1.18 (1.13-1.23)	1.30 (1.22-1.38)	1.22 (1.19-1.26)	1.08 (1.03-1.13)
Other	0.53 (0.36-0.78)	0.35 (0.29-0.43)	0.19 (0.14-0.25)	*0.58 (0.39-0.84)*	*0.36 (0.30-0.44)*	*0.20 (0.15-0.26)*
**Age**						
Early (<46 years-old)	*Ref*	*Ref*	*Ref*	*Ref*	*Ref*	*Ref*
Mid (46-58 years-old)	2.25 (2.13-2.38)	2.08 (2.02-2.14)	1.82 (1.74-1.90)	2.20 (2.08-2.33)	2.00 (1.94-2.05)	1.72 (1.64-1.80)
Late (>58 years-old)	2.08 (1.91-2.26)	2.04 (1.97-2.11)	1.87 (1.78-1.96)	1.81 (1.67-1.97)	1.92 (1.86-1.99)	1.72 (1.64-1.80)
Unknown	1.74 (1.56-1.95)	1.84 (1.75-1.93)	1.42 (1.31-1.53)	1.89 (1.70-2.11)	1.81 (1.73-1.90)	1.42 (1.32-1.53)

© 2023 Nguyen M et al. *JAMA Network Open.*
