# Supplementary material for: Gender, Racial, and Ethnic and Inequities in Receipt of Multiple National Institutes of Health Research Project Grants
Source: JAMA Netw Open. 2023 Feb 28;6(2):e230855. doi: 10.1001/jamanetworkopen.2023.0855 (PMC9975935; doi:10.1001/jamanetworkopen.2023.0855)
Supplement: Supplement 2. — Data Sharing Statement [file jamanetwopen-e230855-s002.pdf]

Data Sharing Statement

Nguyen. Gender, Racial, and Ethnic and Inequities in Receipt of Multiple National Institutes of Health Research Project Grants. *JAMA Netw Open*. Published February 28, 2023. doi:10.1001/jamanetworkopen.2023.0855

Data
Data available: No
